# Structural characterization of human neutralizing antibodies against JC and BK polyomaviruses

**DOI:** 10.1073/pnas.2603048123

**Published:** 2026-07-06

**Authors:** Christina Harprecht, Luisa J. Ströh, Bethany A. O’Hara, Jasmin Freytag, Felix Nagel, Sheila A. Haley, York-Dieter Stierhof, Walter J. Atwood, Thilo Stehle

**Affiliations:** ^a^ Interfaculty Institute of Biochemistry, University of Tübingen, 72076 Tübingen, Germany; ^b^ Department of Molecular Biology, Cell Biology and Biochemistry, Brown University, Providence, RI 02903; ^c^ Microscopy, Center for Plant Molecular Biology, University of Tübingen, 72076 Tübingen, Germany; ^d^ Cluster of Excellence “Controlling Microbes to Fight Infections” (CMFI), University of Tübingen, 72076 Tübingen, Germany

**Keywords:** polyomavirus, neutralizing antibodies, progressive multifocal leukoencephalopathy, structural biology, therapeutic antibodies

## Abstract

The human JC polyomavirus (JCPyV) causes the fatal demyelinating disease progressive multifocal leukoencephalopathy (PML) in immunocompromised individuals. JCPyV frequently undergoes mutations in PML patients, and these variants are thought to establish “antibody recognition holes” that enable the virus to circumvent the antibody response of infected individuals. Many of these PML-associated mutations cluster in the glycan receptor-binding site of the virus. Using X-ray crystallography, we investigated the binding modes of JCPyV VP1-specific human monoclonal antibodies (mAbs) that were isolated from healthy donors and individuals who recovered from PML, and that can recognize a panel of PML-associated JCPyV variants. Our structural analyses show that three out of four of these mAbs bind epitopes that overlap with the glycan–receptor binding site at the surface of the virus particle. The observed interactions explain how PML-associated mutations strategically interfere with antibody recognition, resulting in immune evasion. In contrast, mAb 29B1 engages a region of the capsid that is distant from the glycan site and does not feature mutations associated with PML. The binding site is conserved in the closely related BK polyomavirus (BKPyV), and we show that mAb 29B1 binds both viruses with high affinity and blocks infection. Our findings form an excellent platform for the development of therapeutic Ab approaches and potential vaccination strategies that could protect at-risk patients from infections with both JCPyV and BKPyV. Moreover, small molecules that target the mAb 29B1 binding site could be potentially effective against both viruses.

## Significance

Preventing infectious entry of JC polyomavirus (JCPyV) is a critical strategy for treating and preventing progressive multifocal leukoencephalopathy (PML), a fatal demyelinating disease affecting immunocompromised individuals. This study identifies the binding epitopes of neutralizing antibodies on the major capsid protein VP1 and reveals the structural mechanisms by which these antibodies disrupt viral attachment to host cells. The identification of a distal binding epitope suggests a previously unidentified function for this region, hinting at possible coreceptor binding sites. Additionally, it highlights how mutations within antibody-targeted regions enable immune evasion by the virus. These findings lay the groundwork for the rational design of therapeutic antibody strategies and vaccine candidates targeting JCPyV, while offering potential avenues for cross-protection against the related BK polyomavirus.

Human JC polyomavirus (JCPyV) is a member of the *Polyomaviridae* family. The virus infects approximately 50 to 80% of the adult population and causes mostly asymptomatic, persistent infection in the kidney and urinary tract (1, 2). Upon immunosuppression, JCPyV can be reactivated and spread to the central nervous system (CNS), where it infects glial cells, including astrocytes and oligodendrocytes. JCPyV infection of astrocytes and cytolytic destruction of oligodendrocytes then causes the demyelinating disease progressive multifocal leukoencephalopathy (PML). PML is usually fatal within 1 y of symptom onset, with symptoms including hemiplegia, paralysis, vision loss, and cognitive impairment (3–5). PML affects about 5% of patients carrying the HIV and suffering from AIDS (6–8). However, the incidence of PML has dramatically increased in recent years due to individuals receiving immunosuppressive therapies for autoimmune diseases and immune-mediated diseases such as multiple sclerosis (9–11). To date, no specific treatment for PML is available, and therapy is limited to attempts to reconstitute or restore the immune system. However, such a therapy can result in prominent inflammation of the PML lesion, the so-called immune reconstitution inflammatory syndrome (IRIS). IRIS can cause additional brain damage, and it can also lead to the death of the patient (12–14).

Like all polyomaviruses, JCPyV has a nonenveloped, icosahedral capsid that encloses a circular double-stranded DNA genome of approximately 5 kb. The viral capsid measures about 50 nm in diameter and is formed by 72 pentamers of the major capsid protein VP1. Each VP1 pentamer is associated with a single copy of a minor capsid protein, either VP2 or VP3. VP1 is responsible for viral attachment since its surface-exposed residues mediate interactions with cellular glycan receptors (15, 16). Wild-type JCPyV binds the sialic acid-bearing pentasaccharide LSTc motif in a shallow groove on top of the pentamer to initiate cell attachment (16, 17). Contacts primarily involve the sialic acid moiety of LSTc, which lies in a shallow binding pocket defined by the BC1-, DE-, and HI-loops of one VP1 monomer and the BC2- loop from the neighboring clockwise (cw) VP1 monomer (16) (*SI Appendix*, Fig. S1). This interaction is conserved for the two wild-type strains of JCPyV, Mad-1 (a prototypic neurovirulent JCPyV strain), and WT3 (a strain isolated from the urine of healthy individuals) (17). These two strains differ only in a small number of amino acids outside the receptor binding pocket. Interestingly, however, several studies have identified characteristic mutations of VP1 isolated from the cerebrospinal fluids and blood of PML patients (18–20), and these so-called PML-associated mutations cluster near the binding site for sialic acid (18). The most commonly observed mutations target amino acid positions 54 (e.g., L54F), 266 (e.g., S266F or S266Y), and 268 (e.g., S268F), and all of these mutations adversely affect interactions with LSTc by introducing steric hindrance (18). Several of the remaining PML-associated mutations are also expected to decrease the affinity for LSTc, while still retaining infectivity in a broad range of brain cells. It is still unknown whether these mutations enable the virus to engage an alternate receptor, perhaps located in the brain, instead. However, recent data demonstrate that the mutations enable the virus to evade host immune responses, resulting in a selective advantage (18–21).

The development of prophylactic and therapeutic immunization treatments to clear JCPyV after an infection of the CNS or to avoid the onset of PML is highly desirable. B cells and JCPyV-specific antibodies have been shown to play an important role in the protective function against PML (22). Hence, JCPyV VP1-specific antibodies could guide the development of targeted immunotherapy for PML patients or patients at risk for PML. In a recent study, a panel of JCPyV VP1-specific monoclonal antibodies (mAbs) was isolated directly from donors, including patients who successfully recovered from PML or PML-IRIS (23). In particular, mAbs cloned from memory B cells from a JCPyV-positive multiple sclerosis patient treated with the anti-VLA-4 monoclonal antibody natalizumab (NAT) and who developed PML-IRIS but recovered exhibited exquisite specificity and high affinity toward JCPyV. Ninety % of these studied mAb panel neutralized JCPyV infection in vitro, and a large subset showed reactivity against one or more characteristic PML-associated JCPyV variants. Interestingly, three identified mAbs were also able to bind the human BK polyomavirus (BKPyV), a close relative of JCPyV that is also widespread among the population. Like JCPyV, BKPyV infection is asymptomatic in healthy individuals but can cause severe diseases in immunocompromised patients. In particular, BKPyV can cause polyomavirus-associated nephropathy (PyVAN) in kidney transplant patients and hemorrhagic cystitis in bone marrow transplant recipients (24, 25). Since both diseases can lead to graft loss, the virus poses a serious threat for organ transplant patients.

In order to gain insight into the structural basis of the underlying mechanisms of binding and neutralization of JCPyV and BKPyV, we solved crystal structures of the relevant portions, antigen-binding fragment (Fab) or single chain variable fragment (scFv) molecules, of four potent neutralizing mAbs in complex with JCPyV Mad-1 VP1 pentamers. We furthermore determined the structure of the scFv of one of these (mAb 29B1) in complex with BKPyV VP1. In combination with neutralization assays, these structures explain how antibodies specifically modulate JCPyV neutralization and explain the differential binding toward JCPyV strains and PML mutations. We demonstrate how mAb 29B1 is able to bind various JCPyV PML mutants and moreover, recognize and neutralize BKPyV virions by targeting a conserved epitope that is distant from the receptor-binding surface. Our findings provide an excellent foundation for future vaccine strategies as well as for the use of therapeutic mAbs for the treatment of polyomavirus-associated pathologies. Moreover, our structures inform about target sites for high-affinity small molecules, that could be potentially effective against both viruses.

## Results

### Overall Structures of JCPyV VP1–mAb Fragment Complexes.

To define the basis for mAb binding and neutralization of JCPyV we selected four previously described potent mAbs, 98D3, 27C2, 27C11, and 29B1, that were isolated from a NAT-PML-IRIS patient that recognize the two well-characterized JCPyV wild type strains Mad-1 and WT3 but differ in their interactions with viruses carrying some of the most commonly observed PML-associated mutations (L54F, S266F, S268F) (Table 1 and *SI Appendix*, Fig. S2) (23). We then solved crystal structures of recombinantly expressed Mad-1 JCPyV VP1 pentamers (16) in complex with either Fab (98D3 and 27C2) or scFv (27C11 and 29B1) fragments (*SI Appendix*, Fig. S1 and Table S1).

**Table 1.** Overview of antibody binding profiles regarding JC strains and most common PML mutations

	98D3	27C2	27C11	29B1
Mad-1	+++	+++	+++	+++
WT3	+++	+++	+++	+
L54F	+++	+++	+++	+++
S266F	−	−	+++	++
S268F	−	+++	+++	+++
Neutr.	+	+	+	+
BKPyV	−	−	−	+

Display of binding capabilities of monoclonal antibodies to JC strains Mad-1 and WT3, as well as most prevalent PML mutations L54F, S266F, and S268F. Binding of antibodies to variants annotated with triple indicators was evaluated experimentally and results could further be explained with structural data. Neutralization and BKPyV binding is indicated with a single indicator. These data are based on ref. 23.

**Fig. 1.**
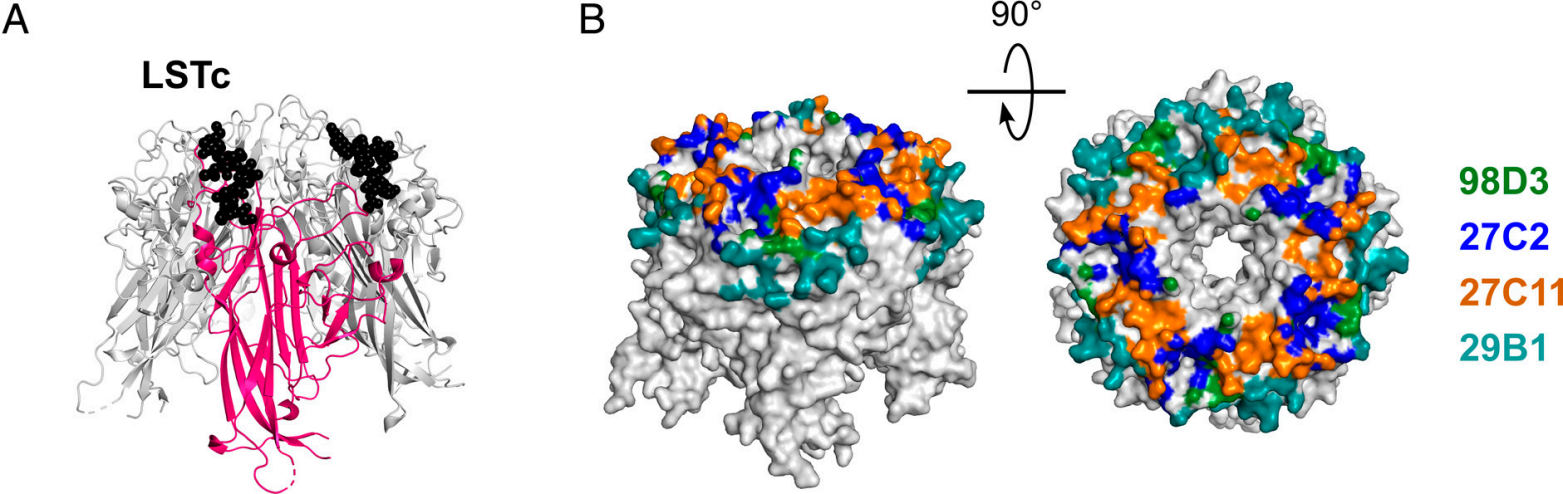
Overview of JCPyV VP1–mAb epitopes. (*A*) Overview of JCPyV VP1 pentamer bound to glycan ligand LSTc (PDB ID 3NXD). The protein is depicted as cartoon with one monomer highlighted in pink. LSTc molecules are represented as black spheres. (*B*) Top and side view overlay of mAb binding sites within a distance of 4 Å of protein residues. The VP1 pentamer is shown in surface representation, and mAb binding regions are highlighted in color (98D3 in dark green, 27C2 in dark blue, 27C11 in orange, 29B1 in teal).

**Fig. 2.**
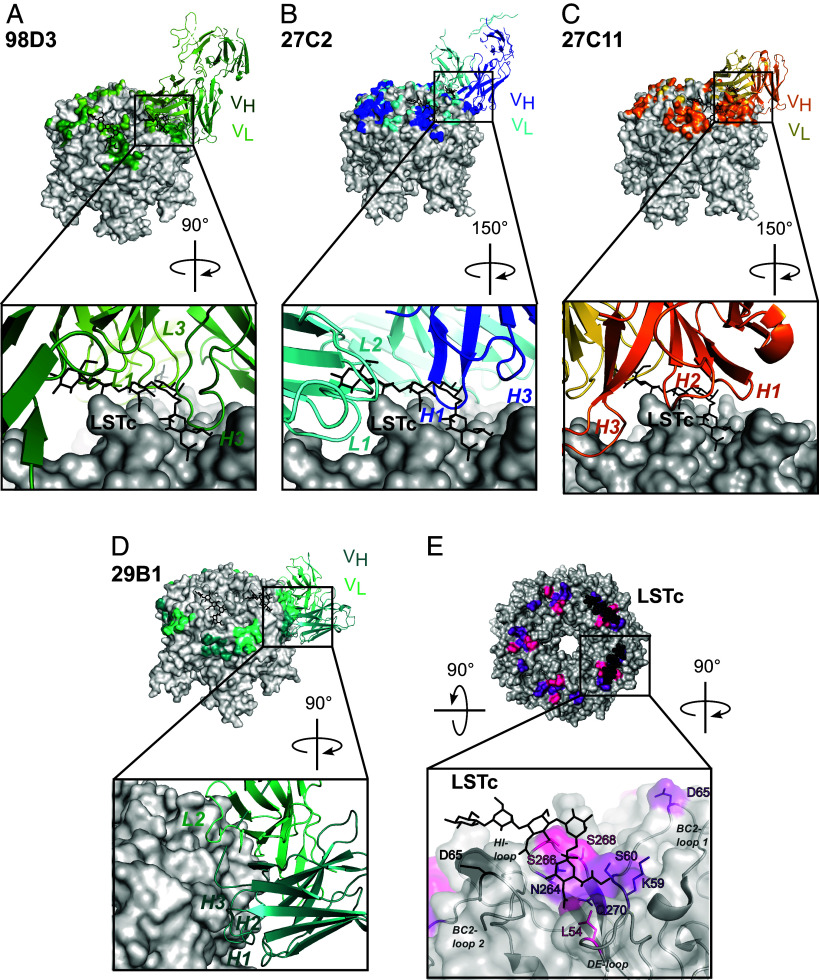
Key interactions of mAbs with JCPyV VP1. Surface representation of JC VP1 pentamers interacting with heavy (V_H_) and light (V_L_) chains of Fabs 98D3 (*A*), 27C2 (*B*) and scFvs 27C11 (*C*) and 29B1 (*D*) in cartoon representation, with glycan receptor LSTc shown in black sticks. The heavy (V_H_) and light (V_L_) chains are shown in darker and lighter colors (green for 98D3, blue for 27C2, orange for 27C11, and teal for 29B1, as indicated by the labels). Constant regions of Fabs 98D3 and 27C2 are not shown (*A* and *B*). Panel *E* shows a surface representation of JC VP1 pentamer with PML mutations highlighted in color. The three residues that are most often mutated in viruses obtained from PML patients are shown in pink (L54F, S266F, and S268F). Residues that less frequently undergo mutations are shown in purple (K59M/E/N, S60P/T, D65H, N264D/T/S, and Q270H). LSTc is shown in black. A close-up view of the LSTc binding site and residues mutated in PML patients is provided underneath.

All four complexes consist of five copies of Fab or scFv molecules bound to a single JCPyV VP1 pentamer, demonstrating that each VP1 monomer establishes identical interactions with a mAb (*SI Appendix*, Fig. S1). This results in a crowded binding arrangement, especially for 98D3 and 27C11, with neighboring copies of the Fab/scFv closely approaching each other. Thus, in a physiologic setting in which complete mAb would bind a virus particle, it is perhaps more likely that only a subset of VP1 monomers in a pentamer will be engaged. The total surface areas buried in the interactions between a VP1 monomer and a Fab (or scFv) range from 707 Å^2^ to 981 Å^2^ (Table 2), which are typical values for antibody/antigen complexes (26). Based on structural comparison with the JCPyV VP1–LSTc complex, the complexes can be divided into two classes: mAbs 98D3, 27C11, and 27C2 engage residues near the top of VP1 in an area that overlaps with the LSTc binding site (16), while mAb 29B1 binds residues at the side of the pentamer, an area that is distant from the LSTc binding site (Figs. 1 and 2).

**Table 2.** Surface interaction area for each mAb

	total	heavy	light
98D3	707 Å^2^	547 Å^2^	160 Å^2^
27C2	975 Å^2^	515 Å^2^	460 Å^2^
27C11	981 Å^2^	849 Å^2^	132 Å^2^
29B1	835 Å^2^	394 Å^2^	441 Å^2^

Values for total areas as well as the areas covered by heavy and light chains are given.

To facilitate the discussion of each complex, complementarity-determining regions (CDRs) CDR1, CDR2, and CDR3 of the mAb heavy chain variable domain (V_H_) are referred to as H1, H2, and H3, respectively, while the corresponding regions of the light chain (V_L_) are named L1, L2, and L3 in the text and in the figures. Interactions involve the BC1-, BC2-, DE-, EF-, and HI-loops of a reference VP1 monomer (pink), and in some cases also loops from cw and ccw neighboring VP1 monomers (gray).

### Effect of PML-Associated Mutants on Antibody Binding to JCPyV.

In order to analyze how the most prominent PML-associated mutations would affect antibody binding, we performed superpositions of the four JCPyV VP1–antibody complexes with previously determined structures of L54F and S268F mutants of JCPyV VP1 (18). Since no structure of the S266F mutant is available, modeling was performed in that case to predict how a phenylalanine side chain at position 266 would affect antibody binding. Less prevalent PML mutations, such as K59M/E/N, S60P/T, are also not structurally characterized, hence modeling was used in these cases to visualize their possible effect on the interactions (*SI Appendix*, Table S2).

### JCPyV VP1 Complex Structures.

The 98D3 epitope spans two neighboring VP1 monomers and overlaps directly with the LSTc binding site (Fig. 2*A*). Key contacts involve the long H3 region, which penetrates deeply into a hydrophobic pocket that normally accommodates the N-acetyl group of the terminal sialic acid moiety of LSTc (Fig. 2*A* and Table 3) (16). Additional contacts, including several hydrogen bonds, are formed between L1 and L3 and the DE- and HI-loops of JCPyV VP1, in between two VP1 monomers (Fig. 2*A* and *SI Appendix*, Fig. S1*A*).

**Table 3.** Overview of key contacts formed by mAbs and JC VP1

mAB	mAB residue	mAb loop	JC VP1 contact	type of formed bond
98D3	F105	H3	F57, F67, F262, L54	hydrophobic cluster
	Y103	H3	N73	hydrogen bond
	N31	L1	R265	salt bridge
	Y32	L1	N123	hydrogen bond
	S93	L3	R265	hydrogen bond
	S93	L3	N130	hydrogen bond
27C2	H101	H3	F57, F67, F262, L54	hydrophobic cluster
	D102	H3	S266	hydrogen bond
	N32	L1	K59	hydrogen bond
27C11	D225	H3	K59	salt bridge
	N227	H3	K59	hydrogen bond
	Y231, Y232, Y233, Y234, Y32, W223	H3	K59, N73, S71	hydrophobic cluster
29B1	D226, D230	H3	K193	salt bridge
	F225	H3	D49, E52, R55	hydrophobic cluster
	Y52	L2	R74	hydrophobic
	Y52	L2	D166	hydrogen bond
	S52	L2	N73	hydrogen bond

Key contacts were identified in crystal structures based on proximity and geometry of the formed interaction based on criteria defined in ref. 27. Loops are named “H” for heavy chains and “L” for light chains of the mAbs.

It was reported that mAB 98D3 is able to recognize the PML-associated mutation L54F but not the S266F and S268F mutants (Table 1). The crystal structure of the 98D3–VP1 complex explains why these mutations would interfere with antibody binding. The two serine residues are part of the binding epitope of 98D3, and replacement of either amino acid with a phenylalanine would lead to direct steric clashes with H1 and H3 (*SI Appendix*, Fig. S3*A*). In contrast, residue L54 is not directly contacted by 98D3 and, therefore, a mutation to the larger phenylalanine could be tolerated at this position. Residue K59, which is sometimes also found to be mutated in PML patients, is directly involved in interactions with residues of H3, thus, a mutation would adversely affect binding of the antibody (*SI Appendix*, Fig. S1*A*). Mutations of the neighboring residue, S60, which is also located at the direct binding interface of JCPyV VP1 and 98D3, would also alter binding of this antibody to JCPyV.

The mAb 27C2 epitope largely overlaps with that of mAb 98D3 but differs in the specific contacts formed. The binding interface spans two neighboring VP1 monomers and compared with 98D3, the contact area is more equally distributed between the 27C2 heavy and light chains (Table 2). Key interactions in the complex are formed by L1 and L2 and H1 and H3 of 27C2 (Fig. 2*B*, Table 3, and *SI Appendix*, Fig. S3*B*). H3 again protrudes into the sialic acid-binding cavity that accommodates the N-acetyl group in the complex with LSTc (16). H3 residues also form several hydrogen bonds with the HI-loop of JCPyV VP1 and the DE-loop of a neighboring VP1 monomer, thus bridging a VP1 subunit interface. One of these contacts is between H3 residue D102 and a PML-mutation hotspot, S266, in the HI-loop. Several H1, L1, and L2 residues are involved in the formation of additional contacts with the BC1- and BC2-loops (*SI Appendix*, Fig. S1*B*).

Antibody 27C2 is capable of binding to the PML-associated mutations L54F and S268F, however, like mAb 98D3, it does not recognize S266F (Table 1). Again, this observation can be rationalized as H3 forms a hydrogen bond with the backbone amide group of S266 (*SI Appendix*, Fig. S1*B*), and the introduction of a bulky phenylalanine side chain would cause steric hindrance with H3 as well as clashes with residues in L2. Since residue K59 forms interactions with 27C2 residues in L1, it is likely that the PML-associated mutations at this position would also adversely affect the antibody binding. Residues N264 and Q270, which are also targeted by PML-associated mutagenesis, are situated at the direct binding interface with H3 of 27C2 (not shown). Substitutions of these residues would therefore most probably lead to diminished binding of mAb 27C2 due to elimination of advantageous interactions.

In case of scFv 27C11, most contacts with VP1 are contributed by the heavy chain (Fig. 2*C* and Table 3). The 27C11 heavy chain CDRs straddle the BC2-loop of JCPyV VP1, with H3 contacting one side of the BC2-loop and H1 and H2 covering the other side. Similar to what was seen in the 98D3 and 27C2 complexes, the H3 region of 27C11 again approaches the sialic acid binding site, although it does not project into the hydrophobic cavity but instead engages surface-exposed residues in the BC2-loop through a network of polar interactions. Some of these residues contact LSTc in the receptor complex, and 27C11 therefore directly interferes with LSTc binding. A cluster of four consecutive tyrosine residues and additional aromatic residues in H3 form extensive contacts with nonpolar regions in the BC2- and the adjacent DE-loop from a ccw monomer (*SI Appendix*, Fig. S1*C*). In addition, H1 and H2 contact residues at the rear of the BC2-loop (not shown). PML mutation hotspot residue K59 in the BC2-loop forms a salt bridge and a hydrogen bond with H3.

All three most frequently occurring PML-associated mutations (L54F, S266F, and S268F) are recognized by mAb 27C11 (Table 1). The crystal structure shows that the binding epitope of 27C11 is shifted away from the sialic acid pocket compared to those of 98D3 and 27C2, with the majority of interactions involving the BC2-loop surface (*SI Appendix*, Fig. S3*C*). In contrast to 98D3 and 27C2, the 27C11 H3 region does not penetrate into the LSTc binding pocket, and as H3 does not directly contact the PML mutation hotspots L54, S266, and S268, the 27C11 antibody can interact with variants that display mutated residues at these positions (Table 1). However, it is likely that other PML-associated mutations such as K59M adversely affect binding of 27C11 as K59 forms a salt bridge with D225 and a hydrogen bond with N227, and mutation of K59 would abolish these favorable interactions. Another BC2-loop residue, S60, is also directly engaging residues of H3 for interactions, which are most likely diminished in PML mutations S60T or S60P. Although the sialic acid-binding site of JCPyV VP1 is not directly contacted by 27C11, the antibody retains neutralizing capacity. The superposition of complexes of JCPyV VP1 bound to 27C11 and LSTc indicates that binding of 27C11 would lead to steric interference with the glucose moiety of LSTc and additional glycan (or protein) residues that would be attached to it in a physiologic receptor (Fig. 2*C*).

Interestingly, scFv 29B1 does not bind to the region involved in LSTc recognition but instead targets a different epitope on the side of the VP1 pentamer that is constituted by the lower portion of the BC2-loop as well as the EF-loops. Light and heavy chains contribute similarly to the binding interface. The H3 packs flat against the VP1 EF-loop and makes extensive contacts. Interactions are mostly polar and involve several hydrogen bonds as well as salt bridges (Fig. 2*D* and Table 3). These contacts are augmented by hydrophobic interactions of H3 with a small crevice formed by hydrophobic portions on VP1. H1 is not involved in any JCPyV VP1 contacts and remains solvent-exposed in the complex. The body of the light chain V_L_ domain and its L1, L2, and L3 loops contact the underside of the BC2-loop and straddle the EF-loop. The epitope does not overlap with the glycan binding site formed by the protruding BC1-, BC2-, DE-, and HI-loops on the outer surface of the pentamer, and thus the 29B1 epitope is not affected by characteristic PML-associated mutations in those loops. The antibody can recognize the three most frequently observed mutations, and it is also expected to bind to all other described PML-mutations such as K59M/E/N, S60P/T, D65H, N264D/T/S, and Q270H. Nevertheless, mAb 29B1 displays neutralizing capacities. The engagement of the bulky antibody by the virus is likely to indirectly block access to the LSTc-bearing receptor, similar to what has been observed in other cases (28).

### Differences in mAb Binding between JCPyV WT3 and Mad-1.

The JCPyV strain WT3, which can be found in the urine of healthy subjects, and the neurovirulent strain Mad-1, are characterized by a few amino acid variations. In order to investigate how these different JCPyV genotypes affect antibody binding, modeling was performed to predict the effect of the amino acid variations between Mad-1 and WT3 on antibody binding.

mAb 98D3 is expected to bind equally well to JCPyV strains Mad-1 and WT3 (Table 1). None of the residues differing between the two strains (R74K, S116T, G133A, L157V, and K163T) would be expected to affect the binding of mAb 98D3 as most of them are distant from the binding epitope. Although residue R74 is situated in the binding interface between JCPyV Mad-1 VP1 and H2 of 98D3, the mutation to a lysine in the case of WT3 does not alter mAb binding.

For antibodies 27C2 and 27C11, none of the differentiating residues between genotype Mad-1 and WT3 is involved in binding nor located in the immediate vicinity of the binding interface, hence the amino acid variations between Mad-1 and WT3 have no effect on binding of 27C2.

Finally, a structural comparison between JCPyV strains Mad-1 and WT3 explains the slightly weaker affinity of mAb 29B1 for JCPyV strain WT3. The introduction of a lysine at position 74 instead of an arginine and a threonine at position 163 replacing lysine in the binding interface reduces the binding capability (Table 1) but does not completely abolish binding of 29B1 to JCPyV WT3.

### Structure of BKPyV VP1 Bound to scFv 29B1.

**Fig. 3.**
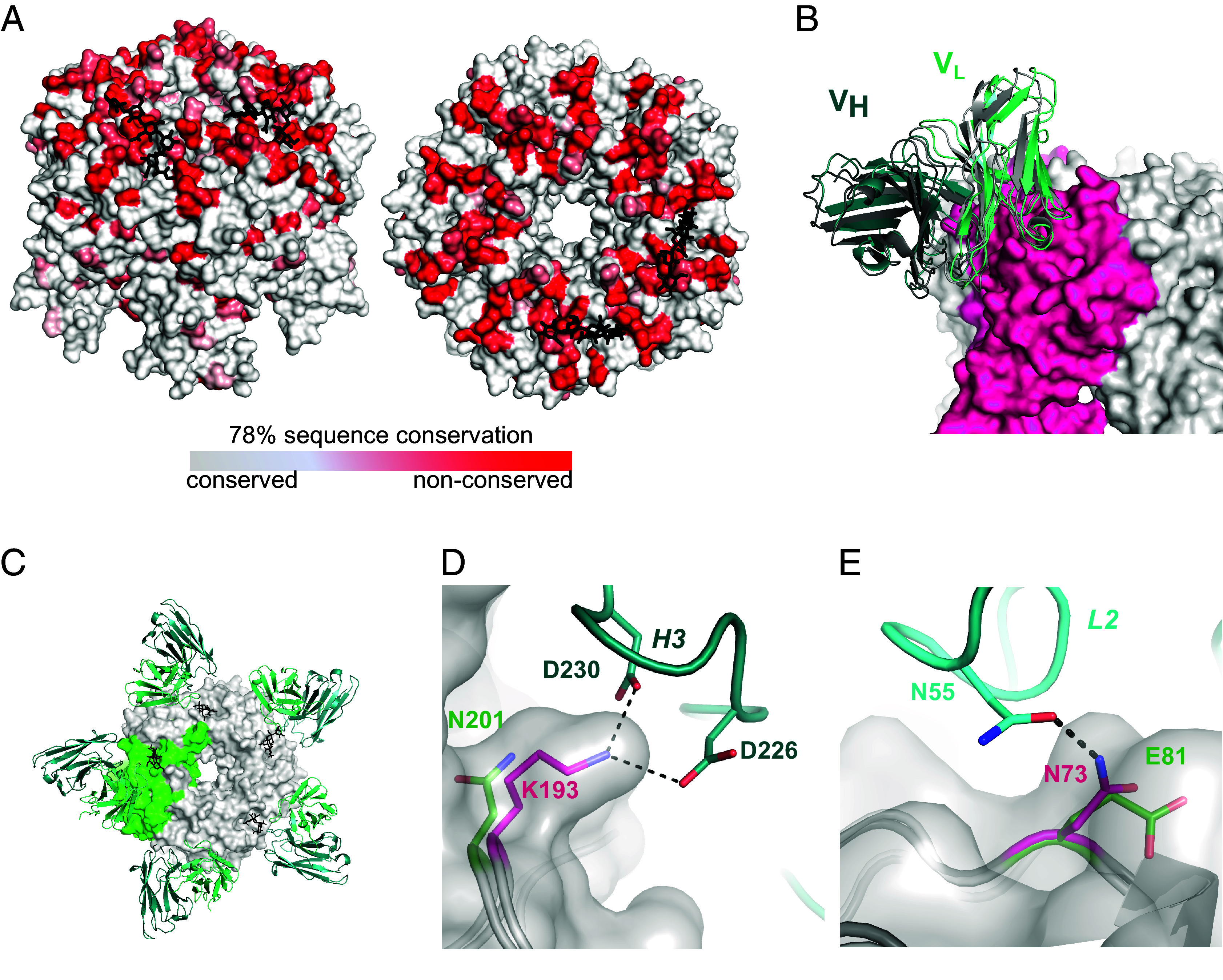
Comparison of 29B1 binding to JCPyV and BKPyV VP1. (*A*) Surface conservation between JCPyV and BKPyV VP1. Surface representation of JCPyV VP1 with residues colored according to their conservation score with gray indicating highly conserved residues and red signifying nonconserved residues. LSTc is shown as black sticks. (*B*) Superposition of scFv 29B1–JCPyV VP1 and scFv 29B1–BKPyV VP1 complex structures, with the JCPyV-bound scFv 29B1 shown in deep teal and the BKPyV-bound scFv 29B1 shown in gray. (*C*) Top view of BK VP1 pentamer (surface representation, gray with one monomer highlighted in green) with the scFvs of 29B1 highlighted in light and dark teal and the glycan receptor GD3 depicted as black sticks. (*D* and *E*) Differences in the binding interfaces between JCPyV and BKPyV VP1. Residue K193 (pink) in JCPyV VP1 interacts with residues D226 and D230 of H3 of 29B1. BKPyV VP1 N201 (green) cannot engage in these interactions. Residue N73 (pink) of JCPyV VP1 is involved in interactions with L2 residue N73. BKPyV VP1 E81 (green) is not engaged in this interaction.

The antibody 29B1 was found to be cross-reactive with BKPyV (genotype I), which shares 78% overall sequence identity with JCPyV VP1 (23). The regions of highest conservation between the two viruses are located on the lateral side of the VP1 pentamer and include the epitope that binds 29B1 in the case of JCPyV (Fig. 3*A*). To determine whether 29B1 is also able to engage this epitope in BKPyV, we determined the crystal structure of BKPyV VP1 in complex with scFv 29B1 at a resolution of 2.6 Å. We found that scFv 29B1 binds to BKPyV VP1 in a manner equivalent to JCPyV VP1 (Fig. 3 *B* and *C*). All observed interactions between scFv 29B1 and BKPyV VP1 are essentially identical to those seen for the JCPyV VP1 complex. When the two VP1 pentamers are superimposed, the rmsd between the bound scFvs in both complexes is only 0.60 Å^2^. The total surface area buried upon scFv 29B1–BKPyV VP1 complex formation is, with 830 Å^2^, identical to the area buried in the JCPyV VP1–scFv 29B1 complex (830 Å^2^, Table 2). Although most interactions are conserved between the two viruses, subtle differences are seen at two positions where JCPyV and BKPyV have nonidentical residues. JCPyV VP1 residues N73 and K193 are replaced with residues E81 and N201, respectively, in BKPyV VP1, but these substitutions only lead to minor changes in interactions (Fig. 3 *D* and *E*). K193 interacts with residues D226 and D230 of H3 in JCPyV, an interaction that is not possible for the N201 side chain in BKPyV.

### Infection Inhibition of mAb Fragments.

**Fig. 4.**
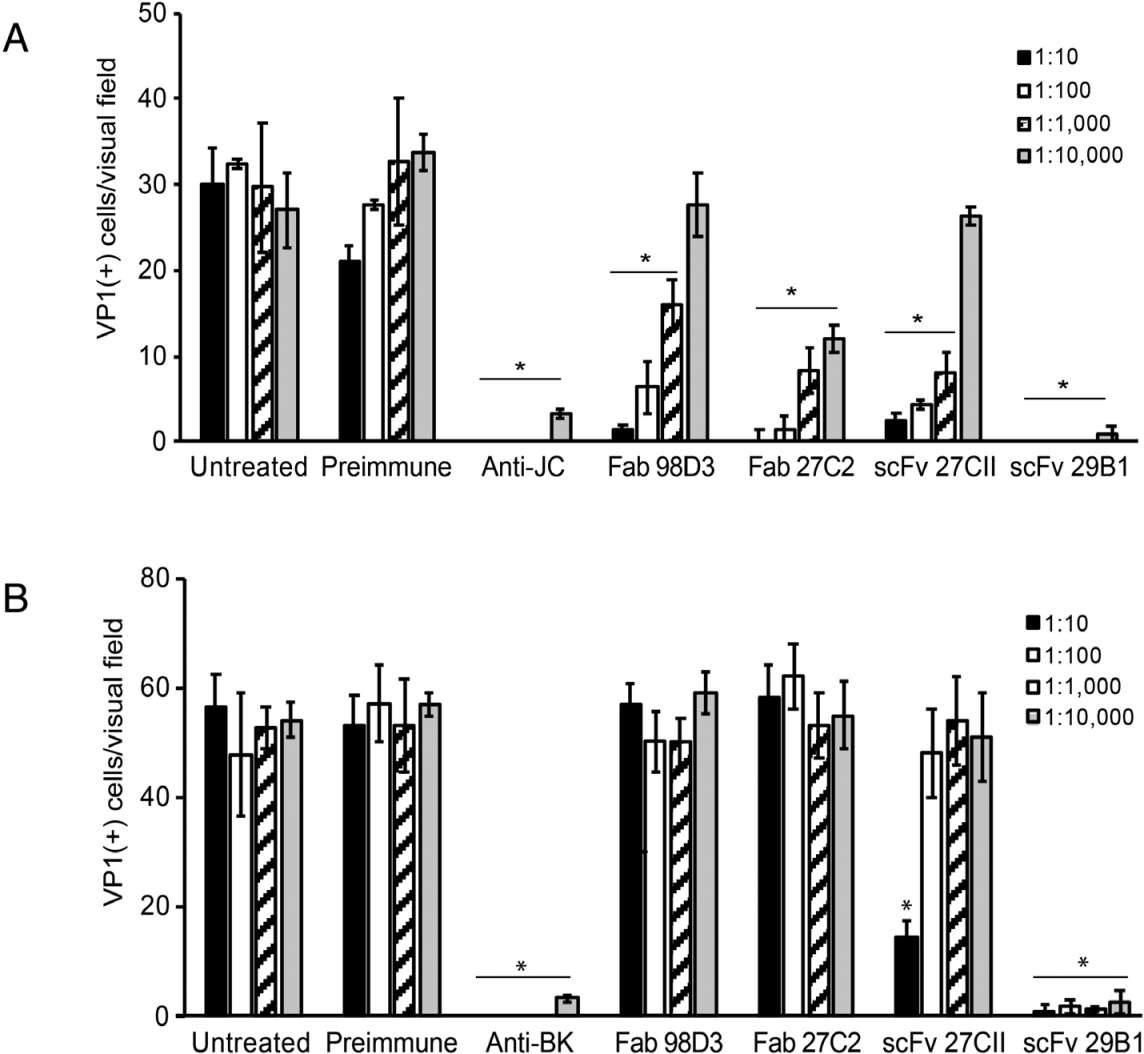
Neutralization of JCPyV and BKPyV infection. (*A*) Inhibition of JCPyV infection by monoclonal antibody fragments. Purified JCPyV was incubated with control sera (preimmune or anti-JC) or with Fab’s as indicated and then used to infect SVG-A cells. Infection was determined by indirect immunofluorescent assay of the late viral protein VP1 at 3 d postinfection. (*B*) Inhibition of BKPyV infection by monoclonal antibody fragments. Purified BKPyV was incubated with control sera (preimmune or anti-JC) or with Fab’s as indicated and then used to infect Vero cells. Infection was determined by indirect immunofluorescent assay of the late viral protein VP1 at 3 d postinfection. *P < 0.05.

Purified JCPyV (Mad-1) and BKPyV (Dunlop) were preincubated with a dilution series of the Fabs 98D3 and 27C2, or with the scFvs 27C2 and 27C11. Incubation of the viruses with rabbit-preimmune sera or rabbit immune sera served as negative and positive controls respectively. The antibody–virus complexes were then incubated with SVG-A cells (JCPyV) or Vero cells (BKPyV) for 1 h at 4 °C. The cells were washed and shifted to 37 °C to initiate an infection. Infected cells were scored by indirect immunofluorescence analysis of viral protein expression (VP1) at 3 d postinfection. Preimmune sera had no effect on infection by either JCPyV or BKPyV, but immune sera directed at both significantly inhibited infection (Fig. 4). The Fabs 98D3 and 27C2 and the scFvs 27C11 and 29B1 all showed neutralization of the viral infection in a dose-dependent manner. 29B1, the only antibody to show cross-reactivity with BKPyV, was the only fragment to neutralize BKPyV infection of Vero cells. As expected, all other antibody fragments did not inhibit BKPyV infection.

## Discussion

The major capsid protein VP1 of JC polyomavirus JCPyV represents the key determinant for viral attachment and cell entry, mediating interactions with sialylated glycans and putatively with the cellular coreceptor 5-hydroxytryptamine type 2 receptor (5-HT_2_R). Here, we report the crystal structures of four neutralizing monoclonal antibodies—98D3, 27C2, 27C11, and 29B1—bound to JCPyV VP1, as well as the complex of 29B1 with BKPyV VP1. These structural insights delineate distinct modes of antibody-mediated neutralization and reveal how the viral surface accommodates or evades humoral immune responses.

Antibodies 98D3, 27C2, and 27C11 share a conserved binding mode, targeting easily accessible epitopes on top of the VP1 pentamer that overlap with the cellular receptor LSTc binding site. This steric interference effectively blocks receptor engagement, explaining the potent neutralization capacities observed for these antibodies. Visualization of these antibody–VP1 complexes reveals that, although complete occupancy of all 360 receptor-binding sites on the capsid is unlikely, even partial coverage would be expected to significantly diminish the overall affinity of the virus for host cell glycans and therefore prevent effective attachment and entry. This mechanism has also been described for BKPyV, where mAb 319C07 mimics glycan receptor GD3, resulting in broad serotype coverage, low risk of viral escape, and high potency, further highlighting receptor blockade as a dominant neutralization mechanism (29).

In contrast, antibody 29B1 binds to a conserved epitope on the lateral surface of the VP1 pentamer, distant from the glycan binding site. This epitope lies in a recessed area between VP1 pentamers, and its engagement suggests an alternative, indirect mode of neutralization. Within the assembled capsid, lateral engagement by 29B1 may impose steric constraints or induce subtle conformational rearrangements required for receptor access, thereby hindering productive interactions with host glycans. Moreover, given that JCPyV infection depends on 5-HT_2_R as a coreceptor, our structural data raise the possibility that 29B1 interferes with coreceptor engagement. It is not known how VP1 engages 5-HT_2_R, but it is conceivable that 29B1 could sterically or allosterically modulate VP1 regions involved in 5-HT_2_R recognition, thereby blocking a postattachment entry step. These findings suggest that JCPyV neutralization can occur via two independent mechanisms: direct blockade of initial attachment or inhibition of subsequent coreceptor-mediated entry.

The cross-reactivity of mAb 29B1 with BKPyV VP1 further highlights its therapeutic potential. Structural analysis revealed that 29B1 binds BKPyV VP1 in a manner nearly identical to its interaction with JCPyV VP1, targeting the conserved lateral epitope. Despite minor differences in residues (e.g., N73 and K193 in JCPyV versus E81 and N201 in BKPyV), the overall binding interface remains intact, preserving neutralization efficacy. Similar binding epitopes have been reported for mAbs 41F17 and MAU868 in the context of BKPyV VP1, although recognizing distinct binding epitopes (*SI Appendix*, Fig. S4) (22, 30). Like 29B1, 41F17 has also been found to exhibit cross-reactivity toward JCPyV VP1, suggesting a conserved PyV immune recognition mechanism by targeting the lateral side of the VP1 pentamers.

The observation that disease-associated VP1 mutations map to the identified antibody epitopes provides further insight into viral immune evasion. Such mutations not only alter glycan-binding preferences but also potentially disrupt antibody recognition, facilitating escape from humoral immunity. These structural adaptations may contribute to the emergence of PML-associated JCPyV variants that exhibit altered receptor tropism and resistance to neutralization.

As antibody-mediated immunity often constitutes the last line of defense against viral infections in immunocompromised individuals, such as patients receiving natalizumab for multiple sclerosis, patients with AIDS-associated T cell lymphocytopenia, or transplant recipients under immunosuppressive therapy (31, 32), understanding the molecular mechanisms that drive mutations has direct clinical relevance. Both JCPyV and BKPyV continue to cause PML and PyVAN, respectively, yet effective antiviral therapies are lacking. The crystal structures presented here provide a molecular framework for the rational design of targeted immunotherapies and vaccines, potentially enabling the development of broadly neutralizing antibodies capable of preventing or treating polyomavirus-associated diseases in several PyVs.

## Materials and Methods

### Recombinant Protein Expression and Purification.

#### JCPyV and BKPyV VP1.

DNA encoding for either amino acids 22–289 of the Mad-1 strain of JCPyV VP1 (UniProtKB entry code P03089) or amino acids 30–300 of BKPyV VP1 (UniProtKB entry code P03088) was cloned into the pET15b expression vector (Novagen) in-frame with an N-terminal His_6_-tag and a thrombin cleavage site. Both proteins were expressed and purified as described previously (16).

#### mAbs.

IgG1 heavy chain expression vector and a κ or λ light chain expression vector encoding for the respective mAbs were cotransfected into HEK 293 cells in a molar ratio of 1:1. mAbs were subsequently purified from the conditioned medium using a protein A column (23).

#### Fabs.

For Fab production, the mAbs 98D3 and 27C2 were dialyzed against 50 mM sodium phosphate buffer, pH 8.0, 1 mM EDTA at 4 °C. After 2 h, the dialysis device with the mAb was transferred into digestion buffer (50 mM sodium phosphate buffer, pH 7.0, 1 mM EDTA, 10 mM cysteine). Papain was prepared as a 2 mg/mL papain agarose solution in digestion buffer and activated by incubation at 37 °C for 10 min. Antibody digestion was performed in a molecular ratio of 6:100 (papain:mAb) and incubated at 37 °C for 5 h. The papain agarose was removed by centrifugation and filtering (0.22 µm). The Fabs were then first separated from the uncleaved mAbs and Fc fractions via a 1 mL protein A column (GE Healthcare) and further purified using a Superdex® 200 Increase 10/300 GL column in 20 mM HEPES, pH 7.5, 150 mM NaCl.

#### scFv 27C11 and scFv 29B1.

For the scFv constructs, the DNA regions encoding the heavy and light chains were amplified separately using primers generating a 15 bp overhang with the target vector sequence and a linker region on the other side and purified by gel extraction. Next, DNA coding for V_L_ and V_H_ chain was linked together via the linker region in a second PCR step using only the outer primers. The amplified DNA was then again purified by gel extraction. The scFv constructs were fused into the respective linearized target vector and transformed into Stellar competent cells (Clontech Laboratories, Inc.) using the InFusion HD Cloning Kit according to the manufacturer’s protocol (Clontech Laboratories, Inc.). scFv 27C11 was cloned into the pET52b vector in the V_L_- (G_4_S)_3_-V_H_ manner including a thrombin site and His_10_ tag at the C-terminus. For scFv 29B1, construct V_L_-(GSTSGSGKPGSGEGSTK)-V_H_ was cloned into the pET47b vector including a HRV 3C protease cleavage site and a His_6_ tag at the N terminus.

The scFv constructs were expressed in SHuffle T7 Express cells (New England Biolabs, Inc.). Precultures were inoculated from single colonies and grown at 30 °C overnight in LB medium containing the respective antibiotic. Main cultures were inoculated and grown to an OD_600_ = 0.6 for induction with 500 µM IPTG. Cells were harvested after expression overnight by centrifugation (9,200× g, 15 min, 4 °C). The cell pellet was resuspended in 100 mM HEPES pH 7.0, 200 mM KCl, 20 mM imidazole for scFv27C11 and 15 mM Tris, pH 6.6, 2% urea; 2.5% glycerol; 1% SDS for scFv 29B1, respectively, using 10 mL buffer per gram of cells. Before lysis via sonification, 1 mM PMSF and Benzonase (MERCK) were added to the resuspended cells. After centrifugation (34,540× g, 35 min, 4 °C), the filtered (0.022 µm), supernatant was loaded onto a HisTrap™ FF crude column (GE Healthcare) and eluted with an imidazole gradient. scFv 27C11 eluted mainly at approx. 800 mM imidazole, while scFv 29B1 eluted at 100 mM imidazole.

Fractions containing scFvs were identified via SDS-PAGE and pooled. In order to remove the His-tag for crystallization purposes, pooled fractions were digested with 10 U thrombin (GE Healthcare) per mg fusion protein scFv 27C11 overnight at 20 °C. 1 mg of HRV 3C protease was added per 10 mg fusion protein scFv 29B1 and incubated overnight at 4 °C. Cleaved protein was separated from uncleaved protein via a second IMAC step and further purified using a Superdex® 200 Increase 10/300 GL column in 20 mM HEPES, pH 7.5, 150 mM NaCl.

### Cells and Viruses.

SVG-A are an SV40 T antigen transformed glial cell line that were derived by subcloning the original and heterogenous population of SVG cells generated by Major and colleagues (33, 34). The SVG-A subclones express the astrocyte specific marker glial fibrillary acid protein (GFAP) and are only 50% related to the original SVG cell culture as determined by Short Tandem Repeat Profiling (34). SVG-A were grown in eagle’s minimal essential medium (MEM) (Corning, Cat# 10-010-CV) supplemented with 10% fetal bovine serum (FBS) (Atlanta Biologicals, Cat# S11150H) and 1% antifungal/antibiotic (Gibco Life Technologies, Cat# 15240-062). Vero cells are African green monkey kidney epithelial cells and were grown in eagle’s minimal essential medium (MEM) (Corning, Cat# 10-010-CV) supplemented with 5% fetal bovine serum (FBS) (Atlanta Biologicals, Cat# S11150H) and 1% antifungal/antibiotic (Gibco Life Technologies, Cat# 15240-062). A lab adapted strain of JCPyV, Mad-1/SVEdelta, was used for SVG-A infections and has been previously described (35). JCPyV was grown in SVG-A cells in a humidified chamber at 37 °C and 5% CO_2_ for 14 d, with the cell culture media replaced at 7 d. For BKPyV infections, the Dunlop strain of BKPyV was purchased from ATCC and propagated in Vero cells in a humidified chamber at 37 °C and 5% CO_2_ for 16 d, with the cell culture media replaced at 7 d. For purification, viral lysates were harvested by scraping cells in the presence of cell culture media. Lysates were frozen at −80 °C and thawed in a 37 °C water bath three times. Deoxycholate was added to obtain a final concentration of 0.25%. Lysates were sonicated three times on ice (50% amplitude 50% duty cycle, power 4, 1 min) and treated with Neuraminidase type II (Sigma, Cat# N6514) at 37 °C for 1 h to release virus from membranes. Samples were subjected to two rounds of VertrelXF (Fisher Scientific Cat #NC9715008) to extract lipids. The viral supernatants were pelleted through a 20% sucrose cushion in a Beckman SW41ti rotor at 150,000× g at 4 °C for 3 h. The viral pellets were resuspended in Buffer A (10 mM Tris-HCl, 50 mM NaCl, 0.1 mM CaCl_2_) and sonicated three times (30% amplitude 50% duty cycle, power 3, 1 min). The resuspended pellets were loaded onto a cesium chloride (CsCl) step gradient (1.29 to 1.35 g/mL) and spun at 115,000× g at 4 °C for 16 h in a Beckman SW55ti rotor. The virus band was isolated and dialyzed against 2 L Buffer A for 48 h, with a buffer change at 24 h.

### Antibodies.

PAB597 is a lab-generated monoclonal antibody directed against SV40 VP1. It was originally developed in the laboratory of Ed Harlow and has since been widely distributed. The antibody cross-reacts with JCPyV and BKPyV VP1. Anti-JC, anti-BK, and preimmune sera were lab generated in our laboratory by injecting rabbits IC with purified JCPyV and BKPyV virions, respectively.

### Neutralization Assay.

SVG-A (JCPyV infections) and Vero (BKPyV infections) cells were plated at 10,000 cells/cm^2^ 24 h prior to infection. CsCl purified JCPyV and BKPyV were pretreated with anti-JC, anti-BK, control preimmune sera, or monoclonal antibody fragments across a linear dilution series for 1 h at 4 °C. SVG-A and Vero cells were shifted to 4 °C for 30 min prior to infection. Cold virus complexes were bound with prechilled SVG-A or Vero cells for an additional hour at 4 °C. Following binding, cells were washed extensively with 1× PBS to remove unbound virus/antibody complexes, shifted to 37 °C and 5% CO_2_, and incubated in complete media as appropriate per cell type.

### Immunofluorescence Analysis.

Three days following infection, SVG-A and Vero cells were fixed using ice-cold 100% methanol at −20 °C for 30 min. Fixed cells were washed in 1× phosphate-buffered saline (1× PBS) and allowed to rehydrate for 15 min at room temperature, followed by incubation with a VP1-specific antibody PAB597 (1:50) in PBS at 37 °C for 1 h. Following primary antibody incubation cells were again washed with 1× PBS followed by incubation with a goat anti-mouse Alexa Fluor 488 conjugated antibody (diluted 1:1,000, Invitrogen, Cat# A11001) in PBS at 37 °C for 1 h. Secondary antibody was removed, cells were washed with 1× PBS and counterstained with DAPI (diluted 1:1,000, ThermoFisher Scientific, Cat# 62248) in PBS for 10 min at room temperature. Infection was scored by indirect immunofluorescent analysis (Nikon E800) for the mean number of VP1(+) cells per field. *P < 0.05, Student’s *t* test. Error bars represent the SD.

### Complex Formation and Purification of VP1 Pentamer–Fab and –scFv Complexes.

Complexes were prepared by mixing purified JCPyV or BKPyV VP1 pentamers with Fabs or scFvs (in approximately sixfold molar excess) in standard buffer (20 mM HEPES, pH 7.5; 150 mM NaCl), followed by incubation of the mixture on ice for 1 h. Complexes were then purified via size exclusion chromatography using a Superdex® 200 Increase 10/300 GL column (GE Healthcare) and the standard buffer. The protein concentrations used for crystallization were determined by measuring the absorbance at 280 nm.

### Crystallization.

Crystals of the Fab 98D3–JCPyV VP1 were obtained at 4 °C using the sitting drop vapor diffusion method with a reservoir solution containing 0.1 M Ammonium tartrate dibasic pH 7.0, and 12% (w/v) PEG 3,350, and a protein solution with a concentration of 6.6 mg/mL. Drops were set up 1:1 (0.3 µL reservoir solution: 0.3 µL protein solution). The Fab 27C2 was crystallized in complex with JCPyV VP1 at 4 °C using the sitting drop vapor diffusion method. The complex was concentrated to 4.8 mg/mL and drops were set up 1:1 (0.3 µL reservoir solution: 0.3 µL protein solution) with a reservoir solution containing 0.1 M Ammonium citrate tribasic pH 7.0, 12% (w/v) PEG 3,350. In case of the scFv 27C11–JCPyV VP1 complex diffracting crystals were grown at 20 °C using the hanging drop method, a reservoir condition containing 100 mM potassium phosphate monohydrate/potassium phosphate dihydrate pH 6.0, 10% (w/v) PEG 8000 and 686 mM NaCl and a protein solution with a concentration of 2.1 mg/mL. 2 µL drops were set up with 1 µL protein and 1 µL reservoir solution. Crystals of the scFv 29B1–JCPyV VP1 complex were obtained at 4 °C using the sitting drop vapor diffusion method with a reservoir solution containing 0.2 M trimethanolamine N-oxide, 0.1 M Tris pH 8.5, 20% PEG 2,000, and a protein solution with a concentration of 2.92 mg/mL. Drops were set up with 0.3 µL reservoir solution and 0.3 µL protein solution. Complexes with BKPyV VP1 were crystallized at 4 °C using the hanging drop method, with a reservoir solution 0.1 M sodium malonate pH 5.5 and 14.4% PEG 3,350. The drops were set up with 1 µL of reservoir solution and 1 µL protein solution with a concentration of 3.5 mg/mL.

For cryoprotection, crystals were transferred into the respective reservoir solution supplemented with 20 % (v/v) glycerol (scFv 29B1), 30 % (v/v) glycerol (Fab 98D3–JCPyV VP1, Fab 27C2–JCPyV VP1) or 30 % (v/v) MPD (scFv 27C11–JCPyV VP1) prior to freezing in liquid nitrogen.

### Structure Determination.

Diffraction data were collected at the SLS beamline X06DA (Villigen, CH) and processed with the XDS package (36) and structures were solved by molecular replacement with (37) within the CCP4 software suite (38). The Fab 98D3- JCPyV VP1 complex structure was solved using search models for a JCPyV VP1 pentamer (PDB code 3NXG) and a Fab which was generated by chainsaw (39) based on the variable regions of light and heavy chains from scFv (PDB code 4KQ3) and Fab (PDB code 5BVJ) structures, respectively. Search models including the JCPyV VP1 pentamer and models obtained from Fab 98D3 using chainsaw were used to solve the Fab 27C2, scFv27C11, and 29B1 complex structures by molecular replacement. Refinement was then performed with Phenix and Refmac5 (40, 41) in an iterative manner with model building done in Coot (42). The final refinement and data statistics are provided in *SI Appendix*, Table S1. Figures were generated using PyMOL (43).

### Modeling of PML Mutants.

For modeling of PML mutants, corresponding residues were altered using Coot (42). Density weighting was reduced and the most probable side chain rotamer was selected. Figures were generated using PyMOL (43).

### Calculation of Surface Area.

To calculate the complex interface areas, the final structures were submitted to the PDBePISA webserver (44).

## Supplementary Material

Appendix 01 (PDF)

## Data Availability

Structural data have been deposited in RCSB PDB (7PA6 (45), 7PA7 (46), 7PA8 (47), 7PA9 (48), and 7PAA (49)).
