# Supplementary material for: Structural characterization of human neutralizing antibodies against JC and BK polyomaviruses
Source: Proc Natl Acad Sci U S A. 2026 Jul 6;123(28):e2603048123. doi: 10.1073/pnas.2603048123 (PMC13367825; doi:10.1073/pnas.2603048123)
Supplement: Supplementary file 1 — Appendix 01 (PDF) [file pnas.2603048123.sapp.pdf]

PNAS

Supporting Information for

Structural characterization of human neutralizing antibodies against JC and BK polyomavirus

Christina Harprecht[1,4#], Luisa J. Ströh[1#], Bethany A. O'Hara[2,5], Jasmin Freytag[1], Felix Nagel[1,6], Sheila A. Haley[2], York-Dieter Stierhof[3], Walter J. Atwood[2], and Thilo Stehle[1*]

[1] Interfaculty Institute of Biochemistry, University of Tübingen, Tübingen, Germany

[2] Department of Molecular Biology, Cell Biology and Biochemistry, Brown University, Providence, RI, USA

[3] Microscopy, Center for Plant Molecular Biology, University of Tübingen, Tübingen, Germany

[4] Current address: BioNTech SE, Mainz, Germany

[5] Current address: Neurotech Pharmaceuticals Inc, Needham, MA, USA

[6] Current address: PROTEROS biostructures GmbH, München, Germany

[#] Contributed equally

*Corresponding author: Thilo Stehle

Email: thilo.stehle@uni-tuebingen.de

This PDF file includes:

Figures S1 to S4

Tables S1 to S2

Figures

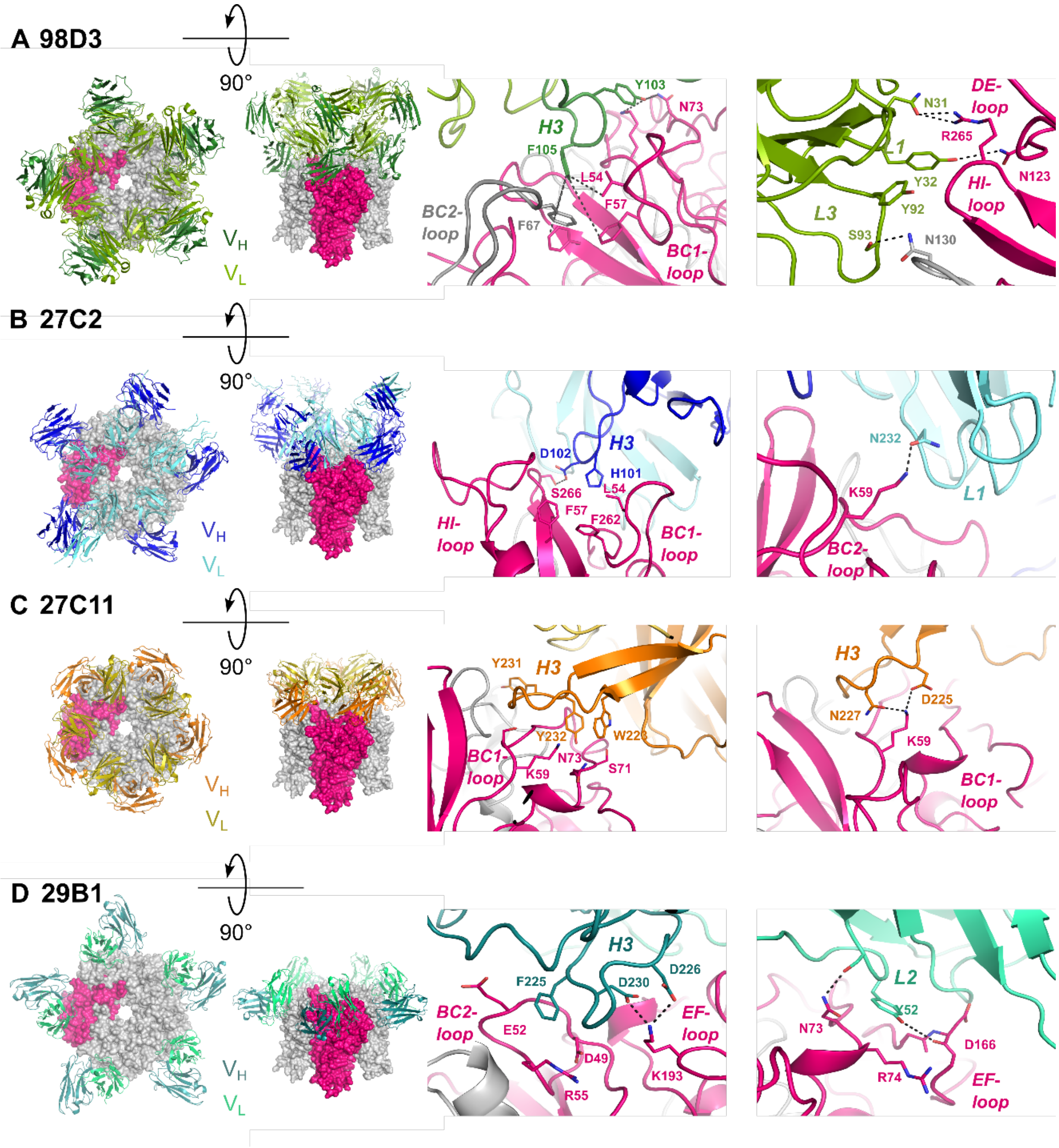

Fig. S1. Overview of JCPyV VP1 – mAB fragment structures and interactions. Left panels: Surface representations of JCPyV VP1 pentamers in top and side view, with one VP1 monomer highlighted in pink and bound Fab 98D3 (A), Fab 27C2 (B), scFv 27C11 (C) and scFv 29B1 (D) shown in cartoon representation. Heavy (V_H) and light (V_L) chains are shown in darker and lighter colors, as indicated by the labels. Constant regions of Fabs 98D3 and 27C2 are not shown. Right panels: Interactions of mAb heavy chains with JC VP1 (pink, cw pentamer in grey) in cartoon representation. Interactions of mAb light chains with JC VP1 (A, B, C, D) and further interactions of the scFv 27C11 H3 loop with JC VP1

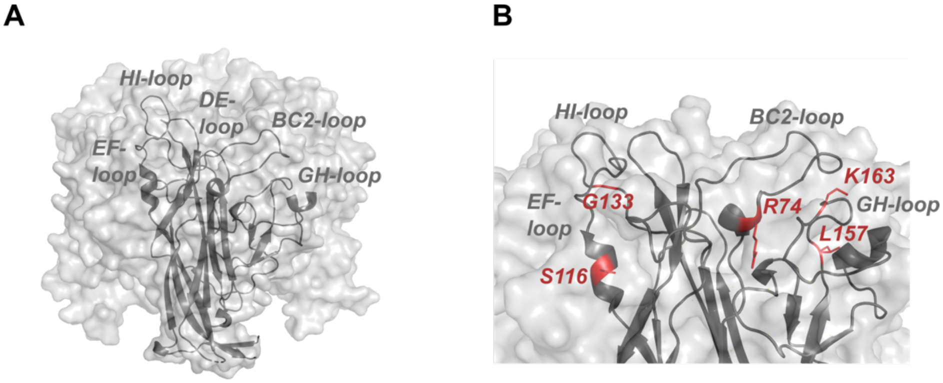

Fig. S2. PML-associated mutations of JCPyV VP1 . (A) Top view of a JCPyV VP1 pentamer in grey surface representation, with one VP1 monomer shown in cartoon representation. (B) Side view of a JCPyV VP1 pentamer in grey surface representation with one monomer shown in cartoon representation. Residues differing between JCPyV strains Mad-1 and WT3 are highlighted in red (R74K, S116T, G133A, L157V and K163T).

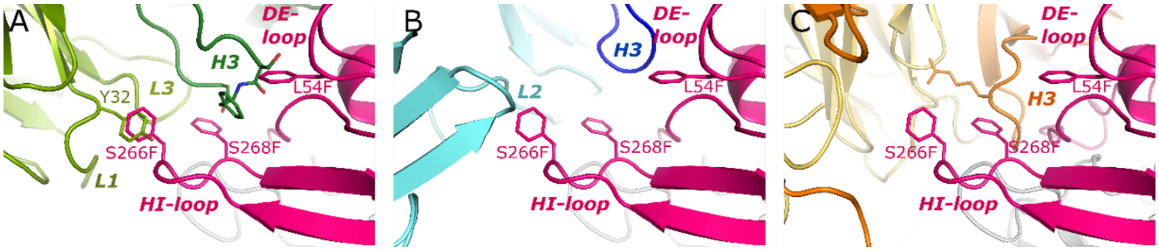

Figure S3. Overview of effects of PML-associated mutations on JCPyV-antibody binding. (A) PML-associated mutations S266F and S268F are not recognized by mAb 98D3 due to clashes between the mutated residues and CDRs L1 and L3 of 98D3, while mutation L54F from the BC1 loop is still recognized. (B) PML-associated mutation S266F is not recognized by mAb 27C2 due to clashes between the mutated residue and L2 of 27C2. (C) Antibody 27C11 is able to recognize all characteristic PML mutations. The introduction of bulky residues does not affect antibody binding.

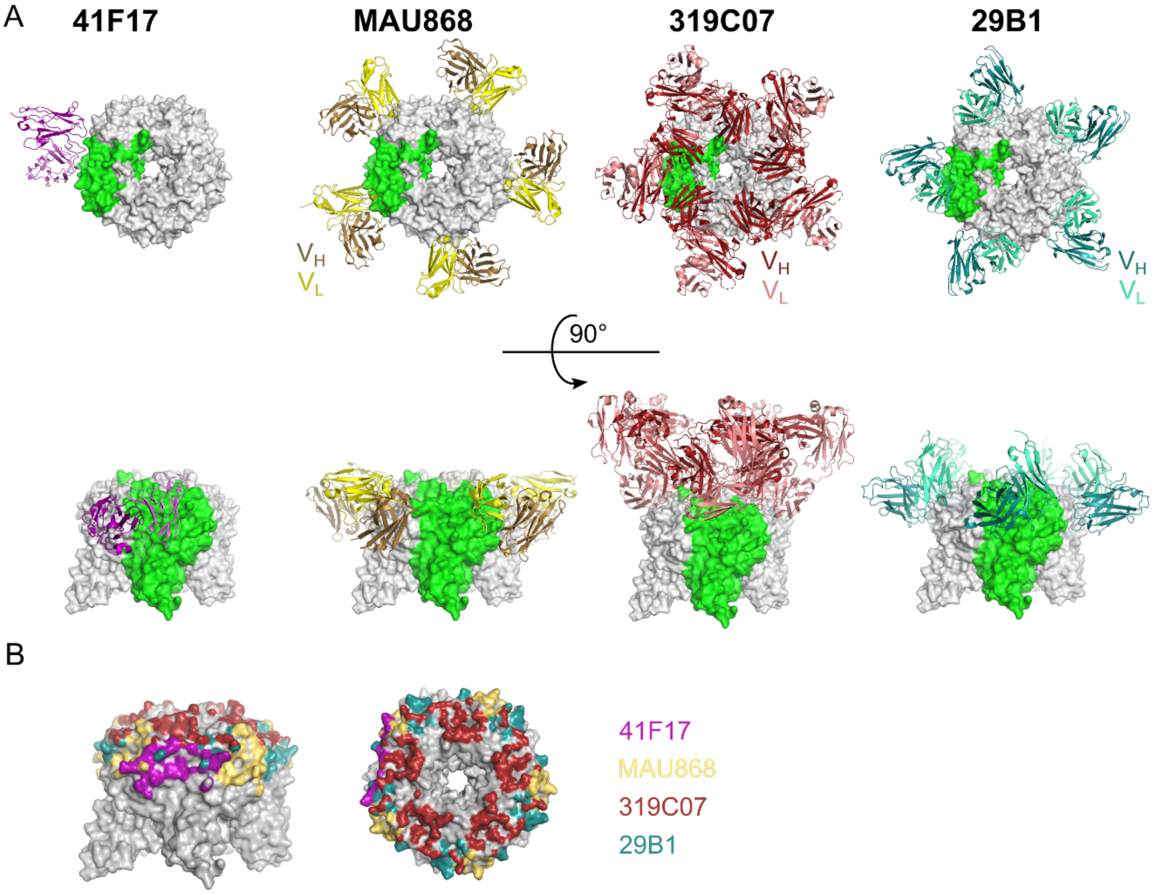

Figure S4. Overview of BKPyV VP1 – mAb fragment structures. (A) Upper panel: Surface representations of BKPyV VP1 pentamers viewed from outside the virion and in side view (turned by 90 degrees) with one VP1 monomer highlighted in green and bound 41F17 (PDB ID 6GGO). MAU868 (PDB ID 8U5L), 319C07 (PDB ID 9RM2) and scFv 29B1 shown in cartoon representation. The heavy (V_H) and light (V_L) chains are shown in darker and lighter colors. Lower panel: Epitopes on VP1. Interactions within a range of 4.0 Å from the mAb fragments are highlighted on the surfaces of the four pentamers. (B) Top and side view overlay of mAb binding sites.

Tables

Table S1. Crystallographic data collection and refinement statistics

PDB ID	**7PA9**	**7PA8**	**7PA6**	**7PAA**	**7PA7**
Data set	**98D3 – JC VP1**	**27C2 – JC VP1**	**27C11 – JC VP1**	**29B1 - JC VP1**	**29B1 - BK VP1 (PDB 7PA7)**
Resolution (Å)	50-2.75 (2.85-2.75)	49.0-3.15 (3.34-3.15)	47.8-1.90 (2.01-1.90)	48.5-3.0 (3.18-3.00)	49.2-2.65 (2.81-2.65)
Space Group	$P2_12_12_1$	$P2_1$	P1	$P2_1$	P1
Unit cell dimensions (Å)	170.97, 202.31, 251.66	137.1, 110.4, 158.2	90.09, 96.11, 190.41	124.1, 172.5, 169.7	114.6, 114.6, 142.3
Unit cell angles (°)	90, 90, 90	90, 93.11, 90	100.91, 93.01, 113.83	90, 104.5, 90	92.3, 106.0, 111.4
Measured reflections	3130828 (239271)	568704 (35387)	1655919 (252959)	916134 (148096)	638907 (94181)
Unique reflections	225685 (16539)	159217 (11220)	431862 (66306)	129715 (20909)	352876 (55527)
Completeness (%)	100.0 (100.0)	99.4 (94.8)	89.9 (85.3)	99.8 (99.5)	94.8 (92.4)
I/σ(I)	11.99 (2.05)	6.6 (1.6)	9.27 (1.15)	10.37 (2.21)	5.9 (1.4)
R_{meas} (%)	28.4 (173.6)	31.0 (111.5)	10.8(119.6)	23.4 (95.7)	17.5 (73.9)
$CC_{1/2}$ (%)	99.5 (68.9)	95.8 (53.7)	99.8 (50.5)	99.1 (72.7)	98.1 (61.8)
Wilson B ($Å^2$)	43.8	43.3	38.8	38.5	34.0
**Refinement**					
R_{work}/R_{free} (%)	20.6/26.2	21.1/24.0	18.3/21.6	23.0/25.0	23.7/26.0
No. of atoms					
Protein	48876	21270	38070	37721	37178
Water	346		2249	218	944
Rmsd bond angle (°)	1.67	1.60	1.60	1.53	1.43
Rmsd bond length (Å)	0.005	0.009	0.010	0.010	0.007
Ramachandran outliers (%)	0.38	0.22	0.31	0.30	0.21
Ramachandran favored (%)	94.51	93.99	92.58	99.70	96.44
Clashscore	5.86	5.32	4.54	4.21	4.01

Table S2. Overview of antibody binding profiles regarding PML mutations. Display of binding capabilities of monoclonal antibodies to PML mutations. Binding of antibodies to variants annotated with triple indicators were evaluated experimentally and results could further be explained with structural data. Single indicators show the effect of PML mutations on antibody binding as evaluated via structural modelling. Data based on reference 23.

	L54F	S266F	S268F/Y	K59M/E/N	S60P/T	D65H	N264D/T/S	Q270H
**29B1**	+++	+++	+++	+	+	+	+	+
**27C11**	+++	+++	+++	-	-	+	+	+
**27C2**	+++	---	+++	-	+	+	+	+
**98D3**	+++	---	---	-	-	+	+	+
